# Genetic study in a family with dopa-responsive dystonia revealed a novel mutation in *sepiapterin reductase* gene

**DOI:** 10.12669/pjms.35.6.1181

**Published:** 2019

**Authors:** Tawfiq Froukh

**Affiliations:** 1 Tawfiq Froukh, Department of Biotechnology and Genetic Engineering, Philadelphia University, Jerash Road, Amman (19392) Jordan

**Keywords:** Intellectual disability, Serotonin, Dopamine, Autosomal recessive, Genome

## Abstract

Dopa-responsive dystonia due to sepiapterin reductase deficiency (OMIM#612716) is caused by recessive mutations in the gene encoding sepiapterin reductase (*SPR*), which plays an important role in the biosynthesis of tetrahydrobiopterin (BH4). One Jordanian patient to first cousin parents is reported in this study. The parents of the proband have recognized the symptoms of their daughter at six months old with motor developmental delay. The symptoms were progressed after-then to include speech delay, seizure, ataxia, oculomotor apraxia, dysarthia and choreoathetosis. Despite of these symptoms, the clinicians in Jordan were unable to diagnose the case. In August 2018, the proband (8 years old) was presented to the department of biotechnology and genetic engineering at Philadelphia University in Jordan for the purposes of performing whole exome sequencing (WES). Analysis of WES data has revealed novel homozygous frameshift variant in the gene *SPR* (NM_003124.4:c.40delG,p.Ala15Profs*100). The variant is heterozygous in the parents and in the healthy male siblings. Therefore, the studied case was diagnosed with sepiapterin reductase deficiency. Because this disease is likely to be treated recommendations were given to the family immediately to start treatments trials. The case in this study illustrates the difficulties of diagnosing sepiapterin reductase deficiency based on clinical symptoms only and thus renders the possibilities of early management. Also, this study reinforces the importance of running WES to undiagnosed neurodevelopmental cases.

## INTRODUCTION

Dystonia, dopa-responsive, due to sepiapterin reductase deficiency (OMIM#612716) is a rare inherited neurotransmitter disorder caused by autosomal recessive mutations in the gene *sepiapterin reductase* (*SPR*). *SPR* is required for the biosynthesis of tetrahydrobiopterin (BH4) which on its turn required for the biosynthesis of serotonin and catecholamine.^1^

This rare disease is underdiagnosed despite the clinical hallmarks of paroxysmal stiffening, oculogyric crises, hypotonia in early stages, cognitive symptoms, speech disturbances, motor delay and dystonic movements in later stages.^2^,^3^ In this study, new mutation in the *SPR* gene using whole exome sequencing (WES) is reported as causative to sepiapterin reductase deficiency. This study stresses the importance of using WES to the undiagnosed neurodevelopmental cases at an early stages for the purposes of better management and possible treatments.^4^

## METHODS

### Family identification

**Fig. 1:**
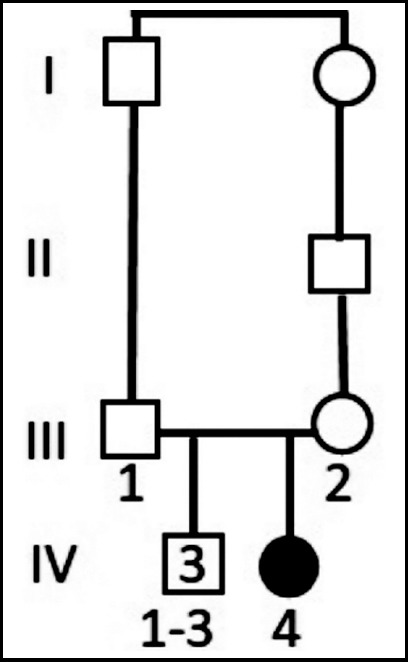
Family pedigree showing the affected daughter (filled circle; IV-4) and the three unaffected male siblings (IV-1-3) born to a first cousin parents once removed (III-1 and III-2).

During the ongoing study of identifying the genetic causes of rare diseases at Philadelphia University in Jordan, a patient with movement disorder was identified. The patient is an eight years old girl (at the time of the study; August 2018) born to a first cousin parents-once removed and has three healthy male siblings (Fig. 1).

### Ethical Approval

This study was approved by the deanship of scientific research and graduate studies at Philadelphia University/Amman-Jordan and informed consents were obtained from all family members.

### Blood sampling

peripheral blood samples were obtained in EDTA tubes from five individuals of the studied family (Fig. 1) including the proband (IV-4), the parents (III-1 and III-2) and two of the male healthy siblings (IV-1 and IV-2).

### Genetic Analysis

DNA was extracted from peripheral blood lymphocytes using Flexi Gene DNA kit. Whole exome sequencing (WES) was conducted to the proband (IV-4) with the following settings: exome capture using Sure Select XT V.6-Post Library Prep Kit was used (Agilent Technologies, USA), exome sequencing using illumina NOVASEQ6000 platform (Illumina Inc., San Diego, CA, USA). The sequencing reads (150 bp pair end) were mapped to the reference genome (UCSC hg19) using the Burrows-Wheeler Aligner software.^5^ Polymerase chain reaction duplicates were removed using samblaster.^6^ Single-nucleotide variants and small insertions/deletions (indels) were called using freebays^7^ and annotated using SnpEff-3.3 (Ensembl-GRCh37.73).^8^ Sequencing was conducted by Macrogen (Seoul, Republic of Korea) and the pipeline megSAP was used.^9^ Only high-quality variants were identified that are located in the protein coding region (according to Ensembl databzase v68) and/or two base pairs flanking splicing sites. Only the variants meeting the following criteria were maintained: (1) at least 20X coverage, (2) mapping quality score≥60, (3) minor allele frequency (MAF)<0.01 in the public (1000 genome, EXAC and gnomAD) and inhouse databases.

The identified variant was confirmed by Sanger sequencing. The primers were designed using primer3 version 4.1.0^10^,^11^ (forward: 5’- GACACCCGTAGCGGTCC-3’ and reverse: 5’- CAGTGCCTCGTCGTTGC-3’). Polymerase chain reaction (PCR) was achieved using Taq polymerase (Invitrogen). The PCR fragments were purified with ExoSAP-IT (Affymetrix Inc.), and sequenced using BigDye™ Terminator V.3.1 cycle sequencing kit and ABI PRISM 3730XL sequencer (Applied Biosystems Inc., USA). Sequences were aligned and analysed using chromas Lite 2.1.1 (Australia Technelusium Pty Ltd).

## RESULTS

### Clinical Data

The parents described the girl’s development story with uneventful pregnancy and delivery, motor delay and seizure. At the age of six months she still could not hold her neck and could not sit. She started to sit independently at two years of age and walking independently at 5 years of age. At the time of meeting the family, the girl showed significant motor abnormalities (ataxia and tremor) and oculomotor apraxia. The parents have presented the following medical reports: (1) motor nerve conduction study and the report mentions abnormal study case in the wrist and ankle, (2) brain MRI and the conclusion was unremarkable, (3) routine 24 channels digital EEG record which showed moderate to high voltage theta activity, (4) blood biochemistry which states high lactic acid (3.5 mmol/L, reference is 0.5-2.2 mmol/L), high asparagine (104 micromol/L, reference 23-79 micromol/L), low creatinine (30 micromol/L, reference 53-123 micromol/L). All of the above medical examinations were done as the girl was three years old. Moreover, the parents have mentioned that their daughter exhibits mild intellectual disability (no IQ test was done).

### Genetic Analysis

**Fig. 2:**
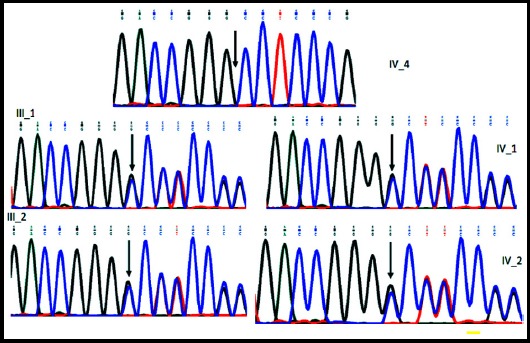
Segregation analysis of the variant NM_003124.4:c.40delG in the gene *SPR* leading to a frameshift mutation indicted by vertical arrow. The affected daughter IV-4 is homozygous for the variant while the parents (III-1 and III-2) and the 2 healthy male siblings (IV-1 and IV-2) are heterozygous for the variant.

The total number of variants that were identified according to the filtering criteria in the methodology is 349 variants. Because the family pedigree suggests an autosomal recessive inheritance pattern, only variants with homozygous genotype were included (14 variants). Of these 14-variants three variants were excluded because they exist as homozygous in the public and in the in-house databases and ten variants were also excluded because they are missense variants predicted as benign and/or tolerated by Polyphen2 Humvar and/or SIFT.^12^,^13^ One variant remained which is one base pair (bp) deletion (chr2:g.73114601delG)in the gene *SPR* (NM_003124.4:c.40delG,p.Ala15Profs*100). The candidate variant was confirmed by sanger sequencing as homozygous in the girl and heterozygous in the parents and in the healthy siblings (Fig. 2).

## DISCUSSION

### Variant Significance

**Fig. 3:**
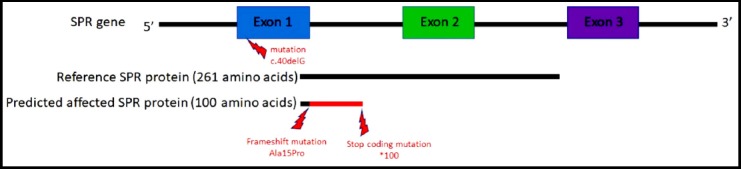
Consequence of the variant NM_003124.4:c.40delG in the gene *SPR* leading a frameshift mutation and its effect on the produced protein length.

The identified variant in this study is novel in the gene *SPR* and is supposed to cause the disease: dystonia, dopa-responsive, due to sepiapterin reductase deficiency (OMIM#612716). It is to note here that the identified variant does not exist in any of the public database neither in the inhouse database. The variant leads to a probably frameshift mutation after amino acid 15 and as a consequence stop coding mutation after amino acid 100 producing a truncated protein (Fig. 3).

### Genotype-phenotype correlation

*SPR* deficiency causes a defect in BH4 synthesis which results in neurologic deterioration due to sever deficiencies of dopamine and serotonin in the central nervous system. The affected individuals show L-DOPA-responsive, diurnally fluctuating movement disorder associated with cognitive delay and neurologic dysfunction. The clinical features of this disorder include: growth retardation, microcephaly, oculogyric crises, oculomotor apraxia, hyperactivity, aggressive behaviour, delayed psychomotor development, mental retardation, dystonia, spasticity, tremor, seizures, dysarthria, axial hypotonia, choreoathetosis, ataxia, hypersomnolence, sleep disturbances and autonomic signs. 14-16

The phenotype of the affected girl in this study overlaps with the clinical features of *SPR* deficiency including: oculogyric crises, dystonia, ataxia, diurnally fluctuating movement disorder, delayed psychomotor development and mild intellectual disability. Differentially, the girl does not exhibit spasticity, microcephaly and growth retardation. In addition, she also sleeps well without disturbances and she is friendly with no aggressive behaviour. This study proposes a novel loss of function variant that leads to a truncated protein as causative of the autosomal recessive dystonia, dopa-responsive, due to sepiapterin reductase deficiency.

## CONCLUSION

This study adds more information to the previous studies and highlights the importance of early genetic testing of the neurodevelopmental diseases in general because early diagnosis might be beneficial for possible treatments. In Jordan few families who are affected with neurodevelopmental disorders undergo molecular diagnosis.^17^-^22^
